# Imaging of Postpartum Ovarian Vein Thrombosis

**DOI:** 10.1155/2012/134603

**Published:** 2012-10-22

**Authors:** Mehmet Bilgin, Osman Sevket, Seyma Yildiz, Rasul Sharifov, Ercan Kocakoc

**Affiliations:** ^1^Department of Radiology, Medical Faculty, Bezmialem Vakif University, 34093 Istanbul, Turkey; ^2^Department of Gynecology and Obstetrics, Medical Faculty, Bezmialem Vakif University, 34093 Istanbul, Turkey

## Abstract

Postpartum ovarian vein thrombosis (OVT) is a rare but serious complication. Clinical findings of OVT are nonspecific. Postpartum OVT, which is a clinically difficultly diagnosed entity, must be thought of in differential diagnosis in cases of postpartum acute abdomen. OVT can be accurately diagnosed by appropriate noninvasive radiologic modalities to start early therapy with anticoagulants and intravenous antibiotics. In this paper, we review the imaging findings of a case with postpartum ovarian vein thrombosis that had been followed up for 6 months by ultrasonography (US), color Doppler US, computed tomography (CT), and magnetic resonance imaging (MRI).

## 1. Introduction

Ovarian vein thrombosis (OVT) is a rare but serious postpartum complication. Clinical findings of OVT are nonspecific; abdominal pain in lower quadrants or right flank pain, fever, and leucocytosis are major findings. Clinical symptoms may mimic especially acute appendicitis and may also resemble many other various clinical situations such as, pelvic infection, ovarian torsion, tuboovarian abscess, hematoma of the broad ligament, and pyelonephritis [1–3].

OVT can cause serious complications such as sepsis, inferior vena cava (IVC) thrombosis, pulmonary thromboembolism, and renal vein thrombosis and they may cause death. Systemic anticoagulation and intravenous antibiotics are the preferred treatment [1–3]. Before the development of cross-sectional imaging methods, OVT was difficult to diagnose. Most cases could be diagnosed at surgery. Since then, the CT, sonographic, and MR imaging findings of this pathology have been well described, and these methods have been shown to be reliable and sensitive for detecting OVT [1, 4–6]. This article presents the imaging features of OVT on ultrasound (US), color Doppler US, computed tomography (CT), and magnetic resonance imaging (MRI) in postpartum period.

## 2. Case Report

A 21-year-old primigravida, who had undergone a normal spontaneous vaginal delivery and Bumm curettage for the rest placenta, presented at 5 days postpartum to our emergency department with severe, stabbing right-sided abdominal and flank pain. She had no nausea, vomiting, anorexia, diarrhea, or fever. Abdominal examination revealed right lower quadrant pain with rebound tenderness.

Laboratory studies showed leucocytosis and anemia (WBC: 13.400/mL, hemoglobin 8.8 g/dL, and hematocrit 26%). The D-dimer result was also positive (640 *μ*g/L; cutoff value: <200 *μ*g/L). The rest of the laboratory studies were within normal values. A diagnosis of acute appendicitis was suspected, and abdominopelvic US examination was performed immediately. 

**Figure 1:**
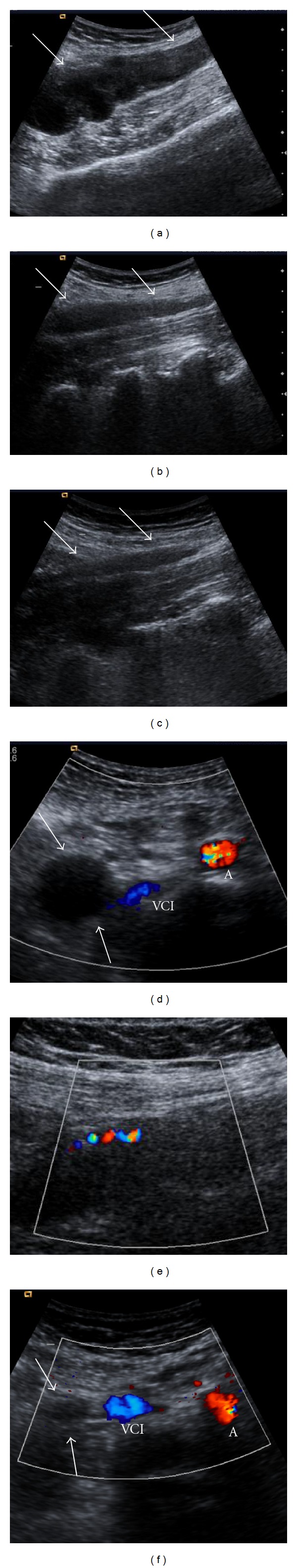
US and color Doppler US images obtained at first day ((a) and (d)), fourth month ((b) and (e)) and sixth month ((c) and (f)) show an enlarged, tortuous noncompressible tubular structure with hypoechoic material centrally, representing thrombosed right ovarian vein, extending superiorly from the right adnexa, lateral to the IVC (white arrows). With time, the width of the vein decreased. At fourth month, color Doppler US image shows flow with recanalization (e). VCI = vena cava inferior, A = abdominal aorta.

On US, appendix vermiformis could not be visualized. The size of the right ovary was minimally increased. US also showed an enlarged (27 × 25 mm), tortuous noncompressible tubular structure with hypoechoic material, representing thrombosed right ovarian vein, extending superiorly from the right adnexa, lateral to the IVC, till to the right renal hilus. On Doppler US, no flow was detected (Figure 1). With these findings, the patient was admitted to our obstetrics and gynecology service and treated with intravenous heparin and antibiotics for right ovarian vein thrombosis.

**Figure 2:**
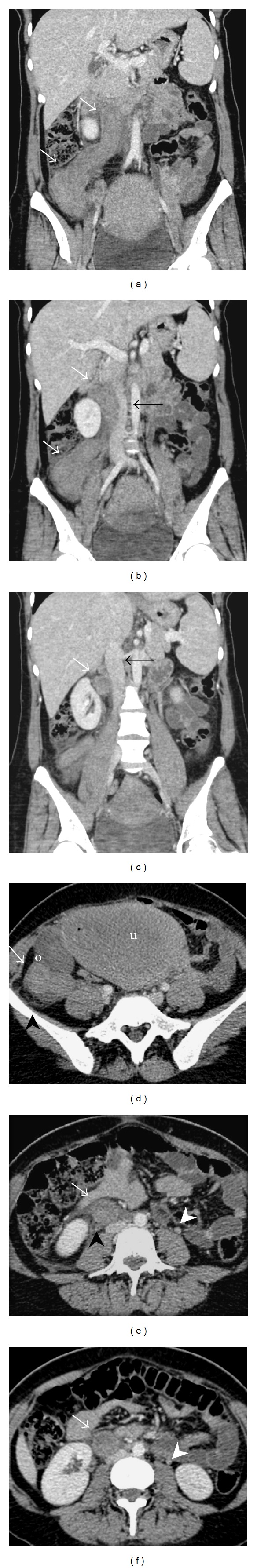
Coronal ((a)–(c)) and axial ((d)–(f)) contrast-enhanced multidetector computerized tomography (MDCT) images demonstrate an enlarged ovarian vein with central hypodensity, representing complete thrombosis (white arrows) extending up to the inferior vena cava, ending at the right renal hilus. There is also surrounding perivascular inflammatory reaction (black arrow heads). Note inflammatory changes in retroperitoneal fat around ovarian vein. Iliac vein is patent. The other ovarian vein is normal in calibration (white arrow heads).

On the first day of the treatment abdominopelvic CT with oral and intravenous contrast material was performed to demonstrate the extension of the thrombus material. CT demonstrated an enlarged right ovarian vein with central hypodensity, representing a complete thrombosis, extending up to the inferior vena cava, ending at the right renal hilus (Figure 2). Inflammatory changes in retroperitoneal fat around right ovarian vein were also present. There was no thrombus in IVC and right renal vein and the left ovarian vein was normal. Normal appearing appendix vermiformis was visualized and there was no evidence of bowel perforation, abscess, diverticulitis, hydronephrosis, or ureteral obstruction.

On the third day of the treatment, the symptoms of the patient were relieved and on the fifth day of the treatment she was discharged with oral 150 mg acetylsalicylic acid and 1 × 0.6 mL (60 mg) subcutaneous heparin (for 5 months) regimen from the hospital. 

**Figure 3:**
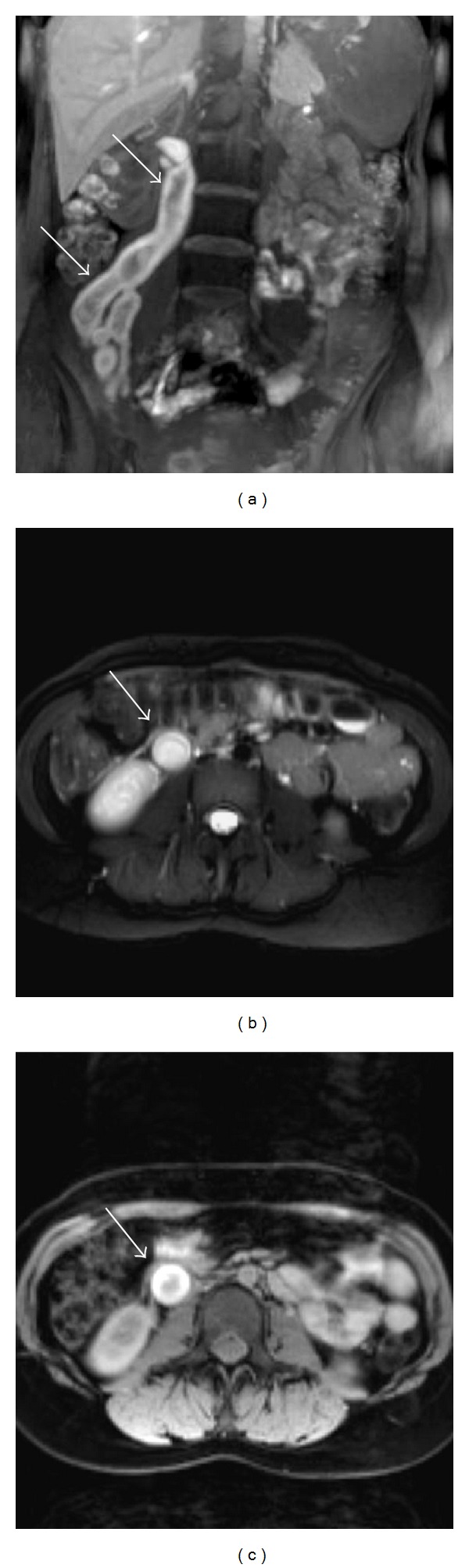
MR images show subacute right ovarian vein thrombosis. Coronal T1-weighted image (a) demonstrates an enlarged right ovarian vein showing increased signal (arrow) with an isointense central focus and axial T2-weighted image (b) shows high signal (arrow) indicative of methemoglobin within a complex thrombus. Contrast-enhanced axial T1-weighted image (c) reveals significant contrast enhancement of the vessel wall.

On the 20th day after the patient discharged from hospital, abdominopelvic MR imaging was performed. MR images showed subacute right ovarian vein thrombosis. T1-weighted images showed increased signal intensity of the enlarged right ovarian vein with an isointense central focus. T2-weighted images showed central high signal. Contrast-enhanced T1-weighted images showed marked contrast enhancement of the wall of the right ovarian vein (Figure 3).

Then, with an interval of one month for 6 months follow-up Doppler US examinations were performed. After the first month the width of the thrombus was gradually decreased and on 6th month the thrombus was measured 8 × 6 mm. After third months color flow consistent with recanalization was seen on color Doppler US. After two months regular menstrual cycle has started.

## 3. Discussion

Ovarian vein thrombosis is a rare but potentially fatal complication of postpartum period, with a reported incidence from 1 : 600 to 1 : 2000 deliveries. OVT occurs 80%–90% in the right side; this could be caused by compression of the right ovarian vein against the sacral promontory due to an enlarged dextroverted uterus and presence of retrograde flow in the left ovarian vein [2, 3]. Although it is not clear, Bumm curettage for the rest placenta may be an extra risk factor and contribute for developing OVT in our case. The severity of this disease is related to the extension of the thrombosis proximally into the inferior vena cava and the risk of pulmonary embolism which develops in 13% of cases with a risk of 4% mortality [4]. Imaging modalities provide a noninvasive and sensitive method of making an accurate diagnosis and can save the patient from surgery. Radiological methods such as US, Doppler US, CT, and MRI are useful in the diagnosis of OVT [2, 3, 7].

On sonography, the thrombosed ovarian vein appears as an anechoic to hypoechoic tubular structure extending superiorly from the adnexa, lateral to the IVC or aorta retroperitoneally [5, 8]. Doppler ultrasound can provide a quick and inexpensive initial examination. On Doppler US, typical findings are absence of color-flow filling and spectral waveform. Sensitivity and specificity of color Doppler US reported relatively low values due to overlying bowel gas which limits the sonographic visualization [9]. On cross-sectional imaging, the diagnosis often is suggested due to the typical appearance of the thrombosed ovarian vein. After a negative or equivocal US, clinical suspicion of ovarian vein thrombosis still persists and cross-sectional imaging modalities are recommended as the next examination [1, 7, 9].

 Characteristic findings on contrast-enhanced CT scans: the thrombosed ovarian vein is visualized as an enlarged tubular retroperitoneal structure, originating in the region of the adnexa and extending cephalad in the retroperitoneal region to the level of the renal veins, representing thrombus and peripheral rim-enhancement of the vein. Secondary signs on CT scans are perivascular inflammatory stranding, an enlarged uterus that contains fluid, and inhomogeneously enhancing parauterine mass believed to be secondary to accompanying pelvic thrombophlebitis. Multiplanar reconstructed coronal images would improve visualization of the IVC thrombus in its entire length, therefore are helpful in evaluating the extent of a thrombus [1, 6, 8].

Since MR is capable of imaging in multiple planes, does not require IV contrast material, and is sensitive to alterations in blood flow, it is of potential value in the diagnosis and follows up of OVT [4]. Because sensitivity of MRI to blood flow and to the paramagnetic effects of iron (in the form of methemoglobin) is superior than CT, differentiation between flowing blood, acute thrombus (less than 1 week old), and subacute thrombus (between 1 week and 1 month) is possible [10]. The variable appearance of the thrombus on spin echo MR sequences is related to the paramagnetic properties of blood degradation products. Generally, the ovarian vein clot is subacute, therefore, on T1-weighted images, the ovarian-vein thrombus showed increased signal intensity, indicative of the presence of methemoglobin. The cause of the central area of relatively lower signal in the ovarian vein is probably related to the complex nature of the thrombus, which may represent a retracted clot [10]. T2-weighted images showed high signal centrally, again indicative of the presence of extracellular methemoglobin. Low-intensity rim was noted peripherally that may have been due to fibrosis or hemosiderin-laden macrophages [4, 10]. MRI is the most reliable investigation with sensitivity as well as specificity of 100% and is recommended in all patients with inconclusive US and Doppler findings [6, 10]. 

İn conclusion, thrombophlebitis of the ovarian vein is a potentially fatal but luckily a rare complication of postpartum. US with Doppler is easy to perform and can accurately diagnose OVT, detect potential IVC involvement, and identify the cephalad extension of IVC thrombus. They can also be used for follow-up of patients being treated with medical treatment. In patients in whom US is suboptimal, due to overlying bowel gas, MRI should be preferred over CT because it does not require intravenous administration of iodinated contrast material and has no risk of radiation, and allows optimal evaluation of the inferior vena cava.
